# Supplementary material for: Bridging Genetics and Precision Medicine in Parkinson’s Disease through GP2
Source: medRxiv. 2026 Mar 27:2026.03.26.26349418. Preprint. [Version 1] doi: 10.64898/2026.03.26.26349418 (PMC13119358; doi:10.64898/2026.03.26.26349418)
Supplement: Supplement 1 [file media-1.docx]

# Supplementary Material

Bridging Genetics and Precision Medicine in Parkinson’s Disease through GP2

Kajsa Atterling Brolin^1,2,†^, Lara M. Lange^3,4,†^, Emily Navarro-Jones^5,6^, Simona Jasaityte^6^, Yuan Ye Beh^7^, Zih-Hua Fang^8,9^, Hirotaka Iwaki^3,9,10^, Lietsel Jones^3,9,10^, Christine Klein^4,11^, Teresa Kleinz^4,12^, Hampton L. Leonard^3,9,10^, Ignacio F. Mata^13^, Alastair J Noyce^2,14^, Njideka Okubadejo^15^, Paula Saffie Awad^16,17^, Laurel Screven^18^, Ai Huey Tan^7^, Marco Toffoli^6^, Dan Vitale^3,9,10^, Andrew Singleton^18^, Cornelis Blauwendraat^18,19^, Mike Nalls^3,9,10,‡^, Huw R Morris^5,6,‡,*^, and the Global Parkinson’s Genetics Program (GP2)

^1^ Translational Neurogenetics Unit, Department of Experimental Medical Science, Lund University, Lund, Sweden.

^2^ Centre for Preventive Neurology, Wolfson Institute of Population Health, Queen Mary University of London, UK.

^3^ Laboratory of Neurogenetics, National Institute on Aging, Bethesda, MD, USA.

^4^ Institute of Neurogenetics, University of Lübeck, Lübeck, Germany.

^5^ UCL Movement Disorders Centre, UCL Queen Square Institute of Neurology, London, UK

^6^ Department of Clinical and Movement Neurosciences, UCL Queen Square Institute of Neurology, London, UK.

^7^ Department of Medicine, Faculty of Medicine, University of Malaya, Kuala Lumpur, Malaysia.

^8^ German Center for Neurodegenerative Diseases (DZNE), Tuebingen, Germany.

^9^ DataTecnica, Washington, DC, USA.

^10^ Center for Alzheimer's and Related Dementias, National Institute on Aging, Bethesda, MD, USA.

^11^ Department of Neurology, University Hospital of Schleswig-Holstein, Lübeck, Germany.

^12^ Section for Movement Disorders, University Hospital of Schleswig-Holstein, Lübeck, Germany.

^13^ Genome Sciences and Systems Biology, Cleveland Clinic Research, Cleveland Clinic, Cleveland, OH, USA.

^14^ Department of Neurology, The Royal London Hospital, Barts Health NHS Trust, London, UK.

^15^ Neurology Unit, Department of Medicine, College of Medicine, University of Lagos, Nigeria.

^16^ Clinica Santa Maria, Santiago, Chile.

^17^ Department of Specialties, Faculty of Medicine, University of Concepción, Concepción, Chile.

^18^ Global Parkinson’s Genetics Program (GP2), Chevy Chase, MD, USA.

^19^ Coalition of Aligning Science (CAS), Chevy Chase, MD, USA.

^†^ Shared first authors.

^‡^ Joint last authors.

### **Survey questionnaire**

1. Parkinson’s disease or other movement disorder drug trials capabilities
   1. My center has previously recruited movement disorder patients to a drug trial.
   2. My center has drug trial infrastructure and we are interested in recruiting participants for drug trials.
   3. My center does not currently have drug trial infrastructure, but we are interested in recruiting participants for drug trials.
   4. We do not have the capacity to be involved in drug trials.
2. Has your site been or do you plan to be involved in an interventional clinical trial for any Parkinsonisn condition between 01/01/2022 and 01/01/2026?
   1. Yes
   2. No
   3. Not sure
3. Please indicate your study code or the name of your GP2 site.
   1. Free text
4. Is your GP2 cohort a single center or a multi-center cohort?
   1. Single center
   2. Multi-center
5. Is your cohort actively recruiting new patients?
   1. Yes
   2. No
6. Which clinical trials for PD or other movement disorders is your site currently participating in?
   1. Free text
7. Would you be happy for GP2 to connect you with pharmaceutical companies regarding potentially trial-eligible participants from your study?
   1. Yes
   2. No
8. Which biomarkers are your site currently collecting for some of your patients?
   1. MRI
   2. PETscan
   3. DATscan
   4. Blood for plasma/serum
   5. CSF
   6. Skin biopsy
   7. Post-mortem brain donation
   8. Smell testing
   9. Other (free text)
9. Which biomarkers would you possibly be able to collect for some of your patients?
   1. MRI
   2. PETscan
   3. DATscan
   4. Blood for plasma/serum
   5. CSF
   6. Skin biopsy
   7. Post-mortem brain donation
   8. Smell testing
   9. Other (free text)
10. Have you generated synuclein seeding assay (SAA) data on CSF for some of your patients?
    1. Yes
    2. No
    3. Unsure
